# Prevalence of latent tuberculosis infection among tuberculosis laboratory workers in Iran

**DOI:** 10.4178/epih.e2017002

**Published:** 2016-12-30

**Authors:** Mahshid Nasehi, Abdolrazagh Hashemi-Shahraki, Amin Doosti-Irani, Saeed Sharafi, Ehsan Mostafavi

**Affiliations:** 1 Center for Communicable Diseases Control, Ministry of Health and Medical Education, Tehran, Iran; 2 Department of Epidemiology and Biostatistics, School of Public Health, Iran University of Medical Sciences, Tehran, Iran; 3 Research Centre for Emerging and Reemerging Infectious Diseases, Pasteur Institute of Iran, Tehran, Iran; 4 Department of Epidemiology and Biostatistics, Pasteur Institute of Iran, Tehran, Iran; 5 Department of Epidemiology, School of Public Health, Hamadan University of Medical Sciences, Hamadan, Iran; 6 Department of Epidemiology and Biostatistics, School of Public Health, Tehran University of Medical Sciences, Tehran, Iran

**Keywords:** Tuberculin test, Latent tuberculosis, Prevalence, Health personnel, Iran

## Abstract

**OBJECTIVES:**

The risk of transmission of *Mycobacterium tuberculosis* from patients to health care workers (HCWs) is a neglected problem in many countries, including Iran. The aim of this study was to estimate the prevalence of latent tuberculosis (TB) infection (LTBI) among TB laboratory staff in Iran, and to elucidate the risk factors associated with LTBI.

**METHODS:**

All TB laboratory staff (689 individuals) employed in the TB laboratories of 50 Iranian universities of medical sciences and a random sample consisting of 317 low-risk HCWs were included in this cross-sectional study. Participants with tuberculin skin test indurations of 10 mm or more were considered to have an LTBI.

**RESULTS:**

The prevalence of LTBI among TB laboratory staff and low-risk HCWs was 24.83% (95% confidence interval [CI], 21.31 to 27.74%) and 14.82% (95% CI, 11.31 to 19.20%), respectively. No active TB cases were found in either group. After adjusting for potential confounders, TB laboratory staff were more likely to have an LTBI than low-risk HCWs (prevalence odds ratio, 2.06; 95% CI, 1.35 to 3.17).

**CONCLUSIONS:**

This study showed that LTBI are an occupational health problem among TB laboratory staff in Iran. This study reinforces the need to design and implement simple, effective, and affordable TB infection control programs in TB laboratories in Iran.

## INTRODUCTION

Tuberculosis (TB) has re-emerged as a major threat to public health, as the spread of multidrug-resistant TB and extensively drug-resistant TB has complicated eradication attempts in recent years [1,2]. Significant geographic variation in TB infection rates persists across the world, which means that health care workers (HCWs) in different areas face different risks of TB [3]. TB is an occupational disease among HCWs, and physicians, nurses, and other HCWs— particularly TB laboratory staff—are at higher risk [4,5]. The relative risk of TB infection in HCWs has been reported to be higher than that of other community groups in all parts of the world [6,7].

TB remains a major public health problem in Iran [1] The prevalence of latent TB infections (LTBI) among HCWs in Iran has been reported to range from 2 to 49% [8-12].

The aim of this nationwide study was to determine the prevalence of LTBI among TB laboratory employees in all mycobacteriology laboratories in Iran.

## MATERIALS AND METHODS

This was a national cross-sectional study performed from October to December 2013 in 50 universities of medical sciences in 31 provinces throughout Iran. All staff members (689 individuals) employed in the TB laboratories of Iranian universities of medical sciences were included as HCWs in this study. Additionally, we included another randomly chosen group of individuals with similar characteristics in terms of sex, age, and length of employment. This group consisted of 317 participants, corresponding to almost half of the TB laboratory staff population from each university of medical sciences. These participants were drawn from the administrative, finance, and service personnel, and were considered to be low-risk HCWs.

A previously designed checklist was used to gather the necessary information regarding the baseline variables. Potential risk factors included work history, length of employment, contact with TB patients, history of smoking, history of purified protein derivative (PPD) testing, Bacillus Calmette-Guérin (BCG) vaccination, and history of TB and other chronic diseases. In addition, we recorded the results of PPD tests.

Chronic disease was defined as morbidity due to hypertension, diabetes mellitus, cancer, or other chronic diseases. Data regarding chronic diseases were obtained based on self-reporting by the participants.

The Ethics Committee of the Pasteur Institute of Iran approved the protocol of this study. All participants enrolled in the study voluntarily and provided written informed consent.

The PPD-S containing 5 tuberculin units (Razi Institute, Tehran, Iran) was injected between the upper one-third and lower two-thirds of the forearm using the Mantoux technique for all participants, and the results were read by an experienced nurse who underwent training regarding the implementation of the Mantoux test in this study. Results were read 72 hours after the injection using the palpation method and a millimeter ruler, and indurations of ≥10 mm were considered positive [13]. For low-risk HCWs who had a positive tuberculin skin test (TST) or clinical signs of active TB, and for all TB laboratory staff, chest X-rays (CXRs) were taken and read by an experienced radiologist at each medical university. Individuals with a positive TST (>10 mm) and any CXR abnormality were examined by a physician specializing in infectious diseases. Active TB was diagnosed based on clinical findings, including general symptoms (such as fatigue, malaise, fever, weight loss, and/or anorexia) and a chronic, productive cough with purulent sputum, in combination with a CXR demonstrating radiological features consistent with TB disease or the detection of acid-fast bacilli in the sputum (smear or culture).

The prevalence of LTBI and the mean diameter of the indurations of the TST test were estimated and reported with 95% confidence intervals (CIs). Analysis of variance was used to compare the mean diameter of the TST indurations across subgroups. Logistic regression was used to identify risk factors associated with positive TST results. Unadjusted and adjusted prevalence odds ra2tios (PORs) were reported to assess the effects of the covariates on LTBI. All statistical analyses were conducted using Stata 11 (StataCorp., College Station, TX, USA) and reported with 95% CIs.

## RESULTS

During the study period, 689 TB laboratory staff and 317 low-risk HCWs were screened for LTBI in 50 medical universities. Males were more common among the study subjects, comprising 68.7% and 71.61% of subjects among the TB laboratory staff and low-risk HCWs, respectively. The mean (standard deviation) age of participants was 38.06 years (7.76 years) and 37.31 years (7.32 years) among the TB laboratory staff and low-risk HCWs, respectively (Table 1). The most common chronic diseases among all participants were hypertension (38.95%) and diabetes (12.63%).

**Figure 1.**
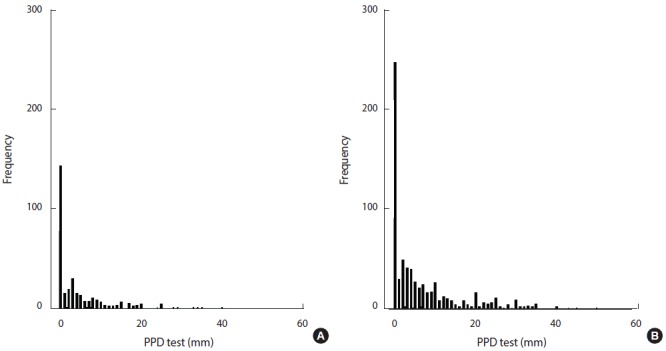
Distribution of TST test among low-risk HCWs (A) and TB laboratory staff (B). TST, tuberculin skin test; TB, tuberculosis; HCW, health care worker; PPD, purified protein derivative.

The distribution of TST indurations in TB laboratory staff and low-risk HCWs is shown in Figure 1. The mean diameter of TST indurations in TB laboratory staff (6.47 mm) was greater than was observed among low-risk HCWs (4.36 mm) (p=0.001). The mean TST induration among TB laboratory staff who had been employed for ≥25 years was significantly larger than was observed staff who had been employed for <1 year (p<0.001). The mean TST induration diameter significantly increased with age (p<0.001). The TST induration diameter among low-risk HCWs was significantly associated with sex (p=0.05), age (p<0.05), and length of employment (p=0.017).

In TB laboratory staff, the mean TST indurations among males (p=0.003) and females (p=0.012) were higher than among low-risk HCWs. In participants 22-31 years of age (p=0.049) and 32-41 years of age (p=0.006), the mean diameter of indurations among TB laboratory staff was much higher than among low-risk HCWs (Table 2).

The prevalence of LTBI among TB laboratory staff was 24.38% (95% CI, 21.31 to 27.74%), which was higher than was observed among low-risk HCWs (14.82%; 95% CI, 11.31 to 19.20%) (p<0.001). Among TB laboratory staff, the prevalence of LTBI increased with age, from 23.91% in subjects 22-31 years of age group to 50% in the oldest age group (Table 3). Inconsistent results were found in the age group of 32-41 years, as the prevalence of LTBI in that group was lower than was observed in the age group of 22-31 years. The prevalence tended to be higher among subjects with low levels of education. The prevalence of LTBI was 51.85% (95% CI, 33.26 to 69.95%) among TB laboratory staff who had been employed for ≥25 years. The prevalence of LTBI among laboratory technicians who had only received a high school diploma (37.93%; 95% CI, 22.15 to 56.76%) was greater than was observed in other job groups. The prevalence of LTBI among TB laboratory staff and low-risk HCWs did not significantly vary according to BCG vaccination status. However, the prevalence of LTBI among vaccinated TB laboratory staff was significantly greater than was observed among vaccinated low-risk HCWs (p=0.001) (Table 3).

Among low-risk HCWs, the prevalence of LTBI increased with age (p=0.006), and was greater in males than in female (p=0.026). The prevalence was higher among subjects with a low level of education. The prevalence of LTBI among low-risk HCWs significantly varied according to length of employment (p=0.009), and the prevalence among individuals who had been employed for ≥25 years (36.00%; 95% CI, 19.59 to 56.50%) was greater than the prevalence among those who had been employed for <25 years (Table 3).

Based on an unadjusted logistic regression analysis, the POR of having an LTBI among TB laboratory staff was 1.85 (95% CI, 1.30 to 2.64) in comparison to low-risk HCWs. The age groups of ≥52 and 42-51 years had PORs of 4.05 and 1.60 in comparison to subjects who were 22-31 years old. The POR of having an LTBI decreased with increasing levels of education. The POR of having an LTBI among TB laboratory staff who had been employed for ≥25 years was 3.70 (95% CI, 1.50 to 9.14) (Table 4).

The adjusted logistic regression analysis revealed that employment as TB laboratory staff (POR, 2.06; 95% CI, 1.35 to 3.17), ≥25 years of employment as TB laboratory staff (POR, 3.15; 95% CI, 1.11 to 8.98), and morbidity due to chronic disease (POR, 1.82; 95% CI, 1.13 to 2.95) were risk factors for having an LTBI (Table 4).

Radiological abnormalities compatible with TB were found in 60 subjects (46 cases among TB laboratory staff and 14 cases among low-risk HCWs). Additionally, 180 sputum samples (3 sputum samples per case) collected from these 60 individuals were subjected to further examination by acid-fast staining, and all samples had negative results. Based on CXRs, physical examination, and acid-fast staining, all these cases were considered to be LTBI.

## DISCUSSION

The results of this cross-sectional study showed that the prevalence of LTBI among TB laboratory staff was significantly greater than among low-risk HCWs. LTBI were two times more prevalent among TB laboratory staff than among low-risk HCWs, and the prevalence increased with length of employment. These results indicate that LTBI may be considered an occupational infection. Age, length of employment, morbidity due to chronic disease, and low levels of education were associated with a greater risk of LTBI.

The risk of transmission of *Mycobacterium tuberculosis* from TB patients to other patients in the hospital and to HCWs has been recognized for many years [14,15]. This risk is greater in areas where TB is endemic, because HCWs must manage larger numbers of TB patients at health care facilities and in TB laboratories [16]. Due to the high global burden of TB and the limited resources of countries in which TB is endemic, health systems must focus mainly on passive case identification and treatment strategies [17,18]. In this situation, even low-cost strategies to reduce TB transmission among HCWs are rarely implemented [16, 19]. Countries where TB is endemic constitute more than 90% of the global TB burden, despite having limited resources [17,18].

This study was the first to survey the prevalence of LTBI among TB laboratory staff, a group of HCWs with a high risk of encountering TB in the workplace in countries where TB is endemic. According to a report of the World Health Organization, the incidence rate of TB in 2015 was 16 (95% CI, 12 to 20) per 100,000 population in Iran [20]. TB laboratory staffs are exposed to sputum samples from active TB patients, and if they do not comply with biosafety regulations, the probability of exposure is increased.

A limited number of the participants of the current study (177 HCWs and 67 non-TB staffs participated in a comparison of TST and interferon-gamma release assay (IGRA) testing. In this study, the prevalence of LTBI identified by TST and IGRA testing was 17% (95% CI, 12 to 21%) and 16% (95% CI, 11 to 21%), respectively [21]. However, we did not have sufficient financial resources to use the IGRA test for all subjects.

The prevalence of LTBI among HCWs has been reported to range from 1.8 to 47.0% in Iran [8,9,19,22]. A low prevalence of LTBI (1.8%) was reported among medical and nursing students in Iran [22]. In contrast, a high prevalence of LTBI (38 to 49%) has been previously reported among HCWs in Iran [8,9,11,12]. These studies were conducted in general hospitals and evaluated LTBI among nurses, ward sisters, physicians, and service workers, most of whom have close contact with patients. The prevalence of LTBI found in the current study among TB laboratory staff is lower than has been reported among other HCWs in Iran [8-12]. A reason for this difference may be attributed to the selection of TB laboratory staff as HCWs in the current study. These HCWs have a risk of TB infection, but have less frequent close contact with patients than nurses, ward sisters, physicians, or service workers. Additionally, they usually work with patient samples in well-equipped laboratories and have adequate awareness of how to handle the clinical samples of suspected TB patients. The prevalence of LTBI in the low-risk HCW population in our study was found to be 14.82%. This may reflect the knowledge and the level of education in low-risk HCWs in our study. Another study showed that a major gap was present among laboratory workers and low-risk HCWs in Iran between knowledge and attitudes, on one hand, and practice on the other hand [23]. Low-risk HCWs in our study with low levels of education had a 33.33% positive TST rate, but increased levels of education were associated with lower rates of LTBI among low-risk HCWs. We believe that the low-risk HCWs in this study were not representative of the general population in Iran, because it seems that they had adequate awareness and knowledge regarding TB. Further study is needed to determine the rate of LTBI among the general population of Iran.

The prevalence of LTBI found in this study was 24.38% among TB laboratory staff, which is significantly lower than has been reported in Ivory Coast (79%), Turkey (72%), Thailand (63%), Peru (63%), Uganda (57%), Brazil (49%), and India (41%) [24-30]. Nurses were the predominant HCW population in most of those studies. Our data help to confirm that LTBI is an occupational disease in HCWs. In addition, many studies have shown the prevalence of LTBI in nurses to be higher than in other HCWs due to close contact with patients [6,19]. Our findings are unusual primarily due to the selection of TB laboratory staff as HCWs. No active TB cases were found in our study. Moreover, little information exists regarding the nosocomial spread of TB among HCWs in Iran. However, TB may be under-reported due to social stigma. Many TB patients have reported facing stigma in the community [31]. It seems that HCWs are less likely to develop active TB because HCWs have a higher average socioeconomic status, and are younger and healthier than the general population in low-income countries [19].

Our study showed that a higher prevalence of LTBI was associated with duration of employment, level of education, and morbidity due to chronic diseases. However, in this study we were not able to assess the associations of LTBI with each type of chronic disease. We categorized all participants into two groups in terms of chronic diseases (yes or no). Hypertension and diabetes were the most common chronic diseases among the participants in this study. Diabetes is a risk factor for active TB, but no consensus exists regarding the effect of diabetes on LTBI [32]. Therefore, our results regarding the association of chronic diseases with LTBI may have been due to the presence of diabetes among the participants. Nonetheless, we recommend that the association of common chronic diseases such as diabetes and hypertension with LTBI be assessed in future studies.

The major limitation of our study is the reliability of the TST method. TST results are influenced by exposure to non-tuberculous mycobacteria, BCG vaccination, type of tuberculin, technique of reading, and the definition of a positive test [19,33]. In Iran, as part of the national immunization program, all newborns receive the BCG vaccine at birth. Therefore, BCG vaccination may have affected the TST results among participants in this study. Despite the above limitations, our results suggest that TB laboratory staff, as HCWs, have a higher risk of TB infection than the estimated risk in the general population.

Our results indicate that the prevalence of LTBI among Iranian TB laboratory staff was high, meaning that the risk of active TB in this occupational group may be greater than in other groups. Therefore, interventional actions such as educational programs about the occupational transmission of TB and the ways it is transmitted are necessary to improve the knowledge of HCWs regarding TB and LTBI, and consequently to reduce the risk of active TB in these groups. Although the available evidence about LTBI among TB laboratory staff is limited, the existing evidence suggests that simple interventions, such as the early diagnosis and treatment of TB patients and the education and training of TB laboratory staff, might be effective for preventing active TB cases. We recommend that in addition to TB laboratory staff, other high-risk groups, such as nurses, primary care HCWs, and clinicians, be screened for LTBI. Another recommendation is a baseline Mantoux test for all TB laboratory staff before the start of employment, with periodic follow-ups to determine if they convert to a positive result.

## Figures and Tables

**Table 1.** Demographic characteristics of TB laboratory staff and low-risk HCWs

Variable	TB laboratory staff	Low-risk HCWs	p-value
Sex			0.25
Female	220 (31.9)	90 (28.4)	
Male	469 (68.7)	227 (71.6)	
Age (yr)	38.06±7.76	37.31±7.32	0.14
Marriage status			0.44
Single	90 (13.1)	36 (11.4)	
Married	599 (86.9)	281(88.6)	
Education (yr)	13.35±7.76	13.14±7.32	0.36
Length of employment (yr)	9.45±7.21	12.73±7.94	0.001
Job			
Expert (laboratory)	221 (32.1)	n/a	
Associate technician (laboratory)	268 (38.9)	n/a	
Technician (laboratory)	29 (4.2)	n/a	
Service personnel (laboratory)	158 (22.9)	n/a	
Administrative laboratory staff	13 (1.9)	n/a	
Administrative staff	n/a	212 (66.9)	
Financial staff	n/a	49 (15.5)	
Service personnel	n/a	56 (17.7)	

Values are presented as number (%) or mean±standard deviation. TB, tuberculosis; HCW, health care worker; n/a, not applicable.

**Table 2.** Comparison of mean TST indurations among TB laboratory staff and low-risk HCW

Variable	TB laboratory staff	p-value	Low-risk HCW	p-value	p-value^1^
Size of TST test result (mm)	6.47 (5.79, 7.14)	-	4.36 (3.60, 5.12)	-	<0.001
Sex					
Male	6.89 (6.04, 7.74)	0.07	4.85 (3.94, 5.75)	0.05	0.003
Female	5.57 (4.49, 6.65)		3.14(1.76, 4.51)		0.01
Age (yr)					
22-31	6.08 (4.81, 7.34)	<0.001	4.12 (2.85, 5.39)	0.01	0.05
32-41	5.35 (4.42, 6.28)		3.16 (2.18, 4.13)		0.006
42-51	7.50 (6.11, 8.90)		6.10 (4.44, 7.76)		0.22
≥52	12.18(6.67, 15.69)		5.57 (0.00, 16.81)		0.33
Marital status					
Married	6.42 (5.69, 7.15)	0.70	4.23 (3.41, 5.05)	0.34	<0.001
Single	6.81 (5.03, 8.59)		5.39 (3.37, 7.41)		0.37
Education					
Elementary	7.95 (5.64, 10.25)	0.15	6.07 (3.40, 8.73)	0.25	0.36
Intermediate	8.48 (4.72, 12.24)		6.00(1.90, 10.10)		0.42
Diploma	7.45 (5.61, 9.30)		4.67 (3.28, 6.07)		0.02
Associate's degree	6.56 (5.43, 6.51)		3.93 (1.92, 5.94)		0.07
License	5.41 (4.31, 6.51)		3.34 (2.34, 5.95)		0.02
Master's or higher	4.80 (1.07, 7.90)		6.43 (0.30, 12.57)		0.61
Length of employment (yr)					
<1	4.61 (2.75, 6.47)	<0.001	4.17 (1.51, 6.83)	0.02	0.87
1-4	6.88 (5.56, 8.21)		3.88 (2.38, 5.40)		0.01
5-9	5.35 (4.23, 6.47)		3.79 (2.10, 5.48)		0.15
10-14	5.09 (3.57, 6.58)		3.00(1.80, 4.81)		0.10
15-19	7.32 (5.32, 9.32)		3.42 (2.02, 4.81)		0.004
20-24	9.50 (6.07, 12.91)		6.72 (4.05, 9.39)		0.21
≥25	12.42 (8.24, 16.61)		7.68 (4.03, 11.32)		0.10
Job					
Expert (laboratory)	5.18 (4.11, 6.26)	0.11	n/a		
Associate technician (laboratory)	6.80 (5.68, 7.91)		n/a		
Technician (laboratory)	8.11 (4.53, 11.69)		n/a		
Service personnel laboratory	7.39 (5.95, 8.82)		n/a		
Administrative staff (laboratory)	7.00 (2.08, 11.92)		n/a		
Administrative staff	n/a		4.61 (3.63, 5.59)	0.52	
Financial staff	n/a		3.37 (1.68, 5.05)		
Service personnel	n/a		4.28 (2.66, 5.89)		

Values are presented as mean (95% confidence interval). TST, tuberculin skin test; TB, tuberculosis; HCW, health care worker; PPD, purified protein derivative; n/a, not applicable.

1 Comparison of the mean size of the PPD test result between laboratory workers and low-risk HCWs.

**Table 3.** Prevalence of LTBIs in TB laboratory staff and low-risk HCWs based on TST results

Variable	TB laboratory staff			Low-risk HCW (%)			p-value^1^
	Prevalence (%)	95% CI	p-value	Prevalence (%)	95% CI	p-value	
Sex							
Male	25.59	21.83, 29.74	0.29	17.62	13.17, 23.17	0.03	0.003
Female	21.82	16.83, 27.79		7.78	3.72, 15.52		0.02
Age (yr)							
22-31	23.91	17.45, 31.77	<0.001	10.39	5.24, 19.54	0.006	0.01
32-41	18.77	14.87, 23.40		9.63	5.65, 15.93		0.01
42-51	29.03	22.93, 35.99		24.50	17.08, 33.85		0.41
≥52	50.00	34.77, 65.22		33.33	2.54, 90.53		0.58
Marital status							
Married	23.87	20.62, 27.46	0.42	14.23	10.59, 18.85	0.41	0.001
Single	27.78	19.46, 37.97		19.44	9.43, 35.86		0.33
Education							
Elementary	38.36	27.88, 50.04	0.006	33.33	18.72, 52.04	0.05	0.63
Intermediate	34.78	18.13, 56.22		25.00	7.81, 56.74		0.55
Diploma	30.53	22.06, 40.55		14.29	8.76, 22.43		0.005
Associate's degree	23.05	18.28, 28.62		11.11	4.63, 24.31		0.07
License	18.43	13.80, 24.18		10.81	6.21, 18.15		0.07
Master's or higher	16.00	6.00, 36.24		14.29	3.39, 44.22		0.89
Length of employment (yr)							
<1	17.78	9.06, 31.92	0.001	16.67	1.85, 67.95	0.009	0.95
1-4	27.68	21.57, 34.76		9.52	4.30, 19.78		0.003
5-9	18.29	13.21, 24.74		11.11	5.34, 21.68		0.19
10-14	17.74	11.95, 25.53		8.33	3.12, 20.43		0.12
15-19	29.03	20.67, 39.11		12.31	6.23, 22.88		0.01
20-24	33.33	21.41, 47.86		25.53	14.99, 40.01		0.40
≥25	51.85	33.26, 69.95		36.00	19.59, 56.50		0.25
BCG vaccination status							
Yes	25.04	21.50, 28.58	0.49	15.26	10.88, 19.64	0.89	0.001
No	22.78	13.46, 32.11		12.76	3.10, 22.44		0.16
Unknown	16.12	2.94, 29.31		12.50	0.00, 37.09		0.79
Job							
Expert (laboratory)	17.65	13.15, 23.26	0.007	n/a	n/a		
Associate technician (laboratory)	23.51	18.80, 28.98		n/a	n/a		
Technician (laboratory)	37.93	22.15, 56.76		n/a	n/a		
Service personnel (laboratory)	32.27	25.42, 39.99		n/a	n/a		
Administrative staff (laboratory)	30.77	11.52, 60.28		n/a	n/a		
Administrative staff	n/a	n/a	-	15.57	11.26, 21.13	0.61	
Financial staff	n/a	n/a		10.20	4.26, 22.50		
Service personnel	n/a	n/a		16.07	8.51, 28.28		
Total	24.38	21.31, 27.74	-	14.82	11.31, 19.20		<0.001

TB, tuberculosis; LTBI, latent tuberculosis infection; HCW, health care worker; TST, tuberculin skin test; CI, confidence interval; BCG, Bacillus Calmette-Guérin; n/a. not applicable.

1 Comparison of the prevalence of LTBI between TB laboratory staff and low-risk HCWs.

**Table 4.** Potential risk factors for LTBI based on TST results

Variables	TST outcome		Unadjusted logistic regression			Adjusted logistic regression		
	PPD+	PPD-	OR	95% CI	p-value	OR	95% CI	p-value
Group								
Low-risk HCWs	270	47	1.00	Reference		1.00	Reference	
TB laboratory staff	521	168	1.85	1.30,2.64	0.001	2.06	1.35, 3.17	0.001
Sex								
Female	255	55	1.00	Reference		1.00	Reference	
Male	536	160	1.38	0.98,1.94	0.06	1.14	0.78, 1.65	0.50
Age (yr)								
22-31	174	41	1.00	Reference		1.00	Reference	
32-41	386	74	0.81	0.53, 1.24	0.34	0.81	0.49, 1.32	0.39
42-51	209	79	1.60	1.05, 2.46	0.03	0.97	0.53, 1.74	0.91
≥52	22	21	4.05	2.04, 8.06	<0.001	1.31	0.54, 3.17	0.55
Education								
Elementary	65	38	1.00	Reference		1.00	Reference	
Intermediate	24	11	0.78	0.35, 1.78	0.56	0.86	0.36, 2.02	0.73
Diploma	156	44	0.48	0.29, 0.81	0.006	0.58	0.34, 1.02	0.06
Associate's degree	237	64	0.46	0.28, 0.75	0.002	0.50	0.28, 0.80	0.005
License	276	52	0.32	0.20, 0.53	<0.001	0.40	0.23, 0.69	0.001
Master's or higher	33	6	0.31	0.12, 0.81	0.02	0.37	0.13, 1.01	0.05
Length of employment (yr)								
<1	42	9	1.00	Reference		1.00	Reference	
1-4	185	55	1.39	0.64, 3.03	0.41	1.45	0.65, 3.23	0.36
5-9	199	39	0.91	0.41, 2.03	0.83	1.01	0.44, 2.32	0.97
10-14	146	26	0.83	0.36, 1.90	0.66	0.90	0.37, 2.19	0.82
15-19	123	35	1.33	0.60, 3.00	0.49	1.53	0.63, 3.67	0.35
20-24	67	28	1.95	0.84, 4.54	0.12	2.04	0.77, 5.35	0.15
≥25	29	23	3.70	1.50, 9.14	0.005	3.15	1.11, 8.98	0.03
Contact with TB patients								
No	451	99	1.00	Reference		1.00	Reference	
Yes	340	116	1.55	1.45, 2.10	0.004	1.32	0.99, 1.58	0.11
History of smoking								
No	690	175	1.00	Reference		1.00	Reference	
Yes	101	40	1.56	1.04, 2.33	0.03	1.18	0.75, 1.86	0.473
Chronic diseases								
No	730	181	1.00	Reference		1.00	Reference	
Yes	61	34	2.24	1.43, 3.53	<0.001	1.82	1.13, 2.95	0.01

TB, tuberculosis; LBTI, latent tuberculosis infection; TST, tuberculin skin test; PPD, purified protein derivative; HCW, health care worker; OR, odds ratio; CI, confidence interval.
